# Patient Experiences With Thyroid Nodules: A Qualitative Interview Survey

**DOI:** 10.1002/oto2.39

**Published:** 2023-03-08

**Authors:** Matthew R. Naunheim, Manuela von Sneidern, Molly N. Huston, Okenwa C. Okose, Amr H. Abdelhamid Ahmed, Gregory W. Randolph, Mark G. Shrime

**Affiliations:** ^1^ Department of Otolaryngology–Head and Neck Surgery Massachusetts Eye and Ear Infirmary Boston Massachusetts USA; ^2^ Department of Otolaryngology–Head and Neck Surgery Washington University in St Louis St Louis Missouri USA; ^3^ Division of Thyroid and Parathyroid Endocrine Surgery, Department of Otolaryngology–Head and Neck Surgery, Massachusetts Eye and Ear Infirmary Harvard Medical School Boston Massachusetts USA; ^4^ Department of Surgery, Massachusetts General Hospital Harvard Medical School Boston Massachusetts USA; ^5^ Department of Global Health and Social Medicine Harvard Medical School Boston Massachusetts USA

**Keywords:** decision‐making, patient preferences, preferences, thematic analysis, thyroid

## Abstract

**Objective:**

To qualitatively explore the broad set of preferences and attitudes patients have about thyroid nodules, which influence the decision‐making process.

**Study Design:**

A descriptive survey design was administered as interviews.

**Setting:**

Outpatient thyroid surgery clinic.

**Methods:**

Semistructured interviews were conducted with 20 patients presenting for initial evaluation of thyroid nodules at a surgeon's office. Probative, open‐ended questions were posed regarding diagnosis, treatment, risk attitudes, and the decision‐making process. Thematic analysis was used to develop code‐transcribed interviews, and an iterative refinement resulted in underlying themes.

**Results:**

During the diagnostic process, patients integrated emotional responses (fear, anxiety, and shock) with rationale concerns (likelihood of cancer, risk assessment), and ultimately relied heavily on expert opinion and recommendation. Contextualization with other personal or familial health problems served as helpful touchstones for decision‐making. Overtreatment and overdiagnosis were not commonly discussed. When thinking about potential therapies, there was a strong bias to action rather than surveillance among patients. Surgical risk and the possibility of lifelong medication, however, were strong motivators for a subset of patients to seek nonsurgical alternatives.

**Conclusion:**

Patients describe a decision‐making process that incorporates emotional response and rational consideration of risks, contextualized within the personal experience and physician expertise. The bias for action and intervention is strong, and most patients strongly weighted physicians' recommendations. Themes from this qualitative analysis may serve as the backbone for future stated preference research pertaining to thyroid disease.

Thyroid nodules are a common clinical problem. Researchers estimate a 2% to 6% prevalence of thyroid nodules on palpation, with approximately 19% to 35% having nodules visible on ultrasound.^1^ Decision‐making regarding thyroid nodules is aided by the Bethesda criteria and American Thyroid Association guidelines, which help define which lesions should be biopsied, followed, and/or treated.^2^, ^3^, ^4^ Decisions are made with multiple factors (size, features, patient factors, etc) taken into account.

Patient preferences should also be assessed for patients with thyroid nodules. To date, patient preferences regarding surgical and observational treatment are not well understood. Incorporating such preferences into shared decision‐making models is not common in head and neck surgery,^5^ but has enormous potential benefit to patients. Many patients present with thyroid nodules for which there are several options available (eg, observation, biopsy, medication, surgery); such conditions are termed “preference sensitive,” and decision‐making should heavily weigh the patient's preference. Incorporation of the patient perspective is critical to ethical medical practice, improves decision‐making, and may improve patient outcomes.^6^, ^7^ Understanding these preferences should begin with a qualitative exploration of thyroid patients' experiences regarding diagnosis and treatment, which underpins further investigation.

The objective of this research is to qualitatively explore the broad set of preferences, concerns, and opinions patients have about thyroid care using structured interviews and thematic analysis. A secondary objective is to develop a list of relevant attributes which are important to patients for future quantitative stated preference research into thyroid disease.

## Methods

### Study Population

This study was approved by the institutional review board of the Mass General Brigham. We recruited a sample of English‐ and Spanish‐speaking patients presenting for evaluation of a thyroid nodule at a specialty otolaryngology clinic from July to September 2020. Patients were included regardless of suspected or confirmed histopathology (eg, papillary thyroid carcinoma, medullary thyroid carcinoma) or stage of disease (eg, locally advanced disease) to reflect the heterogeneity of this population. All patients were seen as referrals from either an endocrinology clinic or a primary care physician's clinic, during which the thyroid nodule had been previously discussed. After a discussion of the patient's condition with the thyroid surgeon, which included education about the nature of the thyroid nodule and potential treatments, contact was made in the clinic by the research assistant informing them of the study. The patient was contacted 24 to 48 hours later for the interview if they agreed to participate. In‐person interviews were not used given contemporaneous restrictions on face‐to‐face interactions during the COVID‐19 pandemic. Patients were limited to adults (18+) and to patients presenting for initial visits; established patients and children were excluded. Patients were selected purposively based on their diagnosis of a thyroid nodule. Other diagnoses (eg, thyroiditis, hypothyroidism, thyroglossal duct cyst) were excluded. All patients had discussed options for surveillance, diagnosis, and treatment, including imaging, fine needle aspiration, and surgical removal as appropriate with their primary surgeon. There was no remuneration.

### Qualitative Interviews

A semistructured interview guide (Supplemental Appendix 1 available online) was developed to query subjects on their experiences with thyroid disease and their opinions of their care related to this. We used published guidelines in the field of thematic analysis and qualitative methods to guide the design of this study.^8^, ^9^ Open‐ended questions were grouped into several topics including thought processes regarding diagnosis, treatment options, treatment side effects, and support during treatment. Specific questions included probed risk appetite and aversion. Additional questions included elicited additional details and were included at the discretion of the interviewer to prompt further discussion. Several yes/no questions were included after the open‐ended questions quantified the frequency of relevant factors in decision‐making across patients. Additionally, the 3 risk‐focused questions from the Jackson Personality Index assessing personality traits were assessed on a 5‐point Likert scale.^10^ We evaluated and refined this guide for clarity and comprehension through pretesting on a convenience sample of providers (n = 3) and patients (n = 2) before final administration.

Interviews were administered by a bilingual (English and Spanish) researcher to 20 patients. Interviews were audio‐recorded.

### Qualitative Analysis

All audio‐recorded tapes were transcribed verbatim, and the transcripts were reviewed independently by 2 independent researchers in accordance with published guidelines for thematic analysis.^8^ We used Taguette, an open‐source software for qualitative data analysis, to catalog the coding scheme.^11^ Data were coded in a deliberately exhaustive fashion and iteratively evaluated for themes regarding the process of diagnosis and treatment of thyroid nodules. An inductive strategy was employed to identify themes and confirm group consensus among the researchers. Themes were mapped diagrammatically to contextualize the data and text excerpted from interviews.

## Results

Twenty semistructured interviews were conducted with patients who presented with thyroid nodules. 65% were female (n = 13), with a median age of 54 (interquartile range 42‐63). A chart review 3 months later indicated that 70% (n = 14) of patients ended up undergoing surgical treatment, and 30% (n = 6) did not. The majority of patients (14/20) indicated that they did not enjoy taking risks, but only 3/20 disagreed with the statement “Taking risks does not bother me if the gains involved are high.” Tables 1 and 2 delineate the topics that emerged from the thematic analysis, grouped into categories pertaining to diagnostic and therapeutic options.

**Table 1:** Themes Arising From the Diagnostic Process

Emotional upset and fear	Cancer concern	Reliance on expertise	Contextualization within past experience
“It was pretty scary, and I thought I was going to throw up and pass out at the same time.” “I mean, I was shocked.” “There's a 1 percent chance that doing the operation that they could hit my vocal cords, and that's, I know its 1 percent but we have no luck in the family so, that 1 percent is like 100 percent to me so I, you know, I get nervous about it.” “I don't like feeling out of control, not knowing what is going on.” “I think I would drop if he said ‘you know you have to get it taken care of immediately.’ Oh man I would've just defecated right there. It was a nice comfortable chair too I didn't want to ruin it.” “Nervous, nervous cause you know, I mean, my first thought is cancer.”	“I am definitely concerned that it has spread farther than they admitted to.” “It was very important to get this treated right away because if it was, if its an aggressive cancer I rather have it treated, you know, quickly.” “So at first, I was told it was not something I needed to worry about, that we'll just keep an eye on it, and that was okay with me until recently when a new doctor said ‘hmm, I don't know about the very smallest of them all, we never checked it out, let me check it out’, and then he came back with 80% malignant and that's when I freaked out.” “I thought it was going to be cancerous and that I was going to die until I did further research on my own, but, but it was scary.” “I'm probably more interested in doing periodic review, you know every 4 months, 6 months and be watching it that way since the doctor told me the one nodule that he's concerned about has about a 30% chance of becoming cancerous.” “I try to be careful and we live in a world filled with all sorts of perils but I try to take measures, I try to do the best choices that I can with diet and exercise and my environment so, as far as a risk of cancer I wouldn't say I'm, I'm not really worried about it, if it happens I'll deal with it but it, it is out there.”	“I've never had a surgery before so it's just trusting doctors to take out my thyroid, is a challenge.” “I formed a strong opinion from my doctor's advice. And then once I was transferred over to a new doctor those came with me.” “It was not a hard decision because anyway we were knowing that because when we consulted a few of the doctors, my primary care physician here, she and also my endocrinology doctor which she, my primary care physician has referred to, she also recommended to have it removed as soon as possible.” “So, we thought that okay, just go with the flow and get it removed so it was not a hard decision as of now.” “I absolutely trust [my doctor] and [my hospital].” “I felt like I really didn't have choice, so that, it was kind of just going down that road.”	“I found out later after talking to some family members that I found out that my mother had a thyroid problem years ago, with nodules, and they were not cancerous, so I was kind of like okay maybe I'm going to follow suit with that, you know that sort of thing.” “My father had cancer as well so, you know, I'm increased chances for getting any type of cancer at this point so when they said that I had nodules it was a little, just, I was taken back.” “I'm BRCA2, I'm always going to be concerned about anything now with cancer, after what I just went through with my breast cancer.””You know they would obviously take out the thyroid not that I would jump up and down for that but, you know, it's not having my leg amputated so I would not be, I would be okay with that.” “[Surveillance is] equal to screenings for breast cancer.” “There's *[sic]* certain cancers that kind of run in the family, you know I've had breast cancer, lung cancer you know things like that so it was like you know, is this, you know is this something that's going to happen to me?”

**Table 2:** Themes Regarding Possible Treatment

Bias to action	Metabolic and medication concerns	Surgical complications	Convenience and financial factors
“Well I did go to see the doctor and they did ultrasound [and] CAT scan and they saw it. And I then had the extractions, the thyroid needle biopsies, came back non‐cancerous, they said the follow up was in a year but that's not cool with me.” “The doctor said that when he gets in there they might have to just, they might just do one half of the thyroid, and then I said to him, I said ‘well gee,’ I said ‘does that mean the nodules on the other side are going to grow? Freaking take them all out.”” “I'm the one that asked him to take the whole thing out so I could put this behind me.” “It was not a hard decision because anyway we were knowing that because when we consulted a few of the doctors, my primary care physician here, she and also my endocrinology doctor which she, my primary care physician has referred to, she also recommended to have it removed as soon as possible.” “I just opted for the surgery because it got rid of it, you know, so.” “I am 100% [decided on surgery] but sometimes I'm just suddenly like only, you know, maybe 2%… If it's only a benign why should I?”	“Nobody wants to not have their thyroid because you know you die if you don't take your medication, so that's pretty nerve wrecking.” “I don't want to say it messes with your metabolism but your body is kind of thrown off from it and you have to figure out how to live without it.” “I was very nervous about that because you know you're taking something out of your body that, you know he says oh you just need a pill and you'll be fine.” “The pills are not going to bother me I take pills, I take enough pills throughout the day anyway so one more isn't going to be a big deal.” “There is a little bit of a concern there, to take a pill every day.”	“Yeah, the only thing with this concerning now is that about my sound, how its going to sound after the surgery, will there be any impact?” “Losing your voice – that's huge.” “I was significantly concerned about effects on any vocal cord damage. As I'm sure it is for everyone, my voice is very important for me so anything that would change that in any way was really scary, I really enjoy singing so anything that could potentially get in the way of that was very scary.” “There's a 1 percent chance that doing the operation that they could hit my vocal cords, and that's, I know its 1 percent but we have no luck in the family so, that 1 percent is like 100 percent to me.” “The biggest thing is the anesthesia and then of course other things that could go wrong but with me” “if we're talking about surgery, I have some skeptical positions on that. I had the open heart surgery and, with all of the surgery and the medication and the, it took me a year to get back. And as a result, I have some vascular dementia. Not terribly bad but, so, but, yeah we have to decide how to treat it when we're going to treat it or whether we are just going to watch it and see what happens.” “Mostly it's the losing your voice, that's huge, I like to talk, we all do, and beyond that breathing is a big deal.” “My thyroid nodule is bigger in size. I was thinking that if I got it treated way before when it was not difficult… it would have not [been] a big scar on my neck. Because now that it is about 11 centimeters, I believe it will be a very big scar.” “I know exactly what the most important is. Pain. That's my only issue, ever.”	“They were recommending few things but I was not following up with that I was busy with my child and all those things which were going on with my work and all that.” “I'm a diabetic I go to the doctor's every three months so I mean you know if I have to go to another doctor the same month or something I'm not that concerned about it.“ “For me also since I am planning to hopefully get pregnant within the next year, earlier would be important for me.” “Originally it was the financial piece. I have some, I have my own insurance from my work but I also just got a full time job in May so it was a little bit of a shock from that.” “Coverage by my insurance would probably be my number one [concern].” “I'm probably more interested in doing periodic review, you know every 4 months, 6 months and be watching it that way since the doctor told me the one nodule that he's concerned about has about a 30% chance of becoming cancerous.”

### Themes Regarding the Diagnosis of Thyroid Nodules

**Figure 1:**
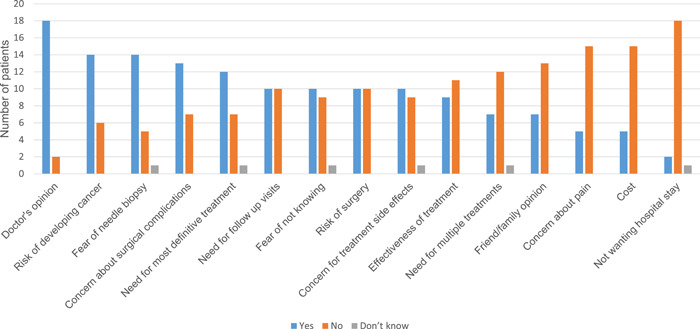
Factors affecting decision‐making, categorical responses (yes/no).

After an iterative review among the authors, several prominent themes emerged regarding the diagnostic process for thyroid nodules (Table 1): emotional reaction to the news; rational concern about cancer; a resulting reliance on expertise; and a contextualization within past experience. An overview of each diagnostic theme is presented below, incorporating both open‐ended (Table 1) and categorical (Figure 1) responses:

a. *Emotional reaction and fear*: Many patients noted that their initial reaction to the discovery of thyroid nodules was primarily emotional. Fear, shock, and anxiety predominated: “It was pretty scary, and I thought I was going to throw up and pass out at the same time” (patient 8). Uncertainty also drove these emotions: “I don't like feeling out of control, not knowing what is going on” (patient 6). Fifty percent (50%) of interviewed patients noted that they had a “fear of not knowing what the nodule is.” While emotions pertaining to possible cancer (see b.) were important, there was also an emotional response to possible treatment modalities. For example, “I think I would drop if he said ‘you know you have to get it taken care of immediately.’ Oh man I would've just defecated right there. It was a nice comfortable chair too I didn't want to ruin it” (patient 4).
b. *Cancer concern*: Fourteen (70%) patients reported that they initially were concerned about the risk of having cancer as evidenced by comments like “I thought it was going to be cancerous and that I was going to die until I did further research on my own” (patient 19) and “I am definitely concerned that it has spread farther than they admitted to” (patient 1). One of these patients, however, noted that the concern for cancer was not all‐consuming: “I try to be careful and we live in a world filled with all sorts of perils but I try to take measures, I try to do the best choices that I can with diet and exercise and my environment so, as far as a risk of cancer I wouldn't say I'm, I'm not really worried about it, if it happens I'll deal with it” (patient 19). Other patients often couched their feelings in terms of likelihoods and percentages. For example, “I'm probably more interested in doing periodic review [for the thyroid nodule]…. since the doctor told me the one nodule that he's concerned about has about a 30% chance of becoming cancerous” (patient 17) and “I was told it was not something I needed to worry about, that we'll just keep an eye on it… and then [the testing] came back with 80% malignant and that's when I freaked out” (patient 16).
c. *Reliance on expertise*: Amidst the emotions and rational concerns about malignancy, patients seemed to rely on—and take comfort in—in their physicians’ opinions and recommendations, with 90% of respondents indicating that they considered their doctor's opinion paramount (Figure 1). “I formed a strong opinion from my doctor's advice. And then once I was transferred over to a new doctor those came with me” (patient 1). Path dependency resulted from such recommendations, from which patients derived significant benefits in the decision‐making process: “So, we thought that okay, just go with the flow and get it removed so it was not a hard decision as of now” (patient 3) and “I felt like I really didn't have a choice, so that, it was kind of just going down that road” (patient 8). Patients generally valued the opinions of their doctors (primary care, endocrinology, surgery), but this did not make the process easy for all patients, with one noting that “I've never had a surgery before so it's just trusting doctors to take out my thyroid, is a challenge” (patient 1).
d. *Contextualization within past experience*: Routinely, patients noted that their decision‐making process revolved around either personal experience or a friend or family member's experience. Sometimes, this was reassuring, for example, “My mother had a thyroid problem years ago, with nodules, and they were not cancerous, so I was kind of like okay maybe I'm going to follow suit with that, you know that sort of thing” (patient 4). Cancer was a significant touchstone in the contextualization process: “There's *[sic]* certain cancers that kind of run in the family, you know I've had breast cancer, lung cancer you know things like that so it was like you know, is this, you know is this something that's going to happen to me?” (patient 4), and “I'm BRCA2, I'm always going to be concerned about anything now with cancer, after what I just went through with my breast cancer” (patient 18). Similarly, surgical experiences served as benchmarks for decision‐making, with 50% of patients responding that the “risk of surgery” was a significant factor for them. “They would obviously take out the thyroid. Not that I would jump up and down for that but, you know, it's not having my leg amputated so I would not be, I would be okay with that” (patient 12).

### Themes Regarding Treatment and Therapeutic Options

Additional themes regarding treatment options included: a bias to action; concern for side effects related to thyroid medication; fear of surgical complications; and factors related to convenience and finance (eg, hospital stay, frequency of follow‐up). An overview of each treatment‐related theme is presented below, incorporating both open‐ended (Table 2) and categorical (Figure 1) responses:

a. *Bias to action*: Most patients demonstrated their preference to “get it out” even if that led to the possibility of overtreatment. “The doctor said that when he gets in there they might have to just, they might just do one half of the thyroid, and then I said to him, I said ‘Well gee… Does that mean the nodules on the other side are going to grow? Freaking take them all out’” (patient 4). To many this seemed a simple decision: “I'm the one that asked him to take the whole thing out so I could put this behind me” (patient 5), and “I just opted for the surgery because it got rid of it” (patient 7). Nonetheless, many had doubts about the recommended course of action, based on the possibility of uncertainty: “I am 100% [decided on surgery] but sometimes I'm just suddenly like only, you know, maybe 2%… If it's only a benign why should I?” (patient 1); “I then had the extractions, the thyroid needle biopsies, [they] came back non‐cancerous, they said the follow up was in a year but that's not cool with me” (patient 2).
b. *Metabolic and medication concerns*: Apart from surgical concerns (see c.), concerns about medications and the hormonal effect of treatment were repeatedly broached by patients. One noted “Nobody wants to not have their thyroid because you know you die if you don't take your medication, so that's pretty nerve wrecking” (patient 16), and another noted about hypothyroidism that “I don't want to say it messes with your metabolism but your body is kind of thrown off from it and you have to figure out how to live without it” (patient 18). Some were more bothered by taking medication than others, with one patient noting “The pills are not going to bother me I take pills, I take enough pills throughout the day anyway so one more isn't going to be a big deal” (patient 4) while another patient who was naïve to medication noted that “There is a little bit of a concern there, to take a pill every day” (patient 14).
c. *Surgical complications*: 65% of patients reported that surgical complications factored into their decision‐making about their thyroid nodule, and education about voice change after thyroid surgery (both from providers and the Internet) weighed on patients' minds; one patient noted “I was significantly concerned about effects on any vocal cord damage. As I'm sure it is for everyone, my voice is very important for me so anything that would change that in any way was really scary, I really enjoy singing so anything that could potentially get in the way of that was very scary” (patient 9). Another couched this again in terms of percentages: “There's a 1% chance that doing the operation that they could hit my vocal cords, and that's, I know it's 1% but we have no luck in the family so, that 1% is like 100% to me” (patient 4). Concerns were not limited to voice. Risks related to anesthesia, mental status changes, pain, and scarring were also noted. For example, “My thyroid nodule is bigger in size. I was thinking that if I got it treated way before when it was not difficult… it would have not [been] a big scar on my neck. Because now that it is about 11 centimeters, I believe it will be a very big scar” (patient 3).
d. *Convenience and financial factors*: Categorical responses indicated that cost, hospital stay, and insurance coverage were of less concern overall (Figure 1); however, several patients were vocal about these. Treatment for a likely benign nodule was delayed in one patient due to these factors: “They were recommending a few things but I was not following up with that. I was busy with my child and… my work” (patient 3). Follow‐up surveillance was noted to be mildly inconvenient but also provided reassurance.

## Discussion

The present study explored patients' feelings, experiences, and preferences as they pertain to the diagnosis and treatment of thyroid nodules. Patients described the confluence of emotional response with rational consideration of cancer and treatment‐related risks, considering heavily both past personal experience and input from physicians. There was a strong bias to action, with the specter of cancer looming large in patients' minds, but concomitantly considerations about surgical risks, hormonal disruption, and long‐term factors related to medication and convenience emerged. Certainly, knowing these factors can help physicians guide patients, and they can also serve as touchstones for educational interventions and future quantitative analysis (state preference research, risk tradeoff assessment) of patient preferences related to thyroid disease.

These data are illustrative in the context of current thyroid nodule management. Despite the existence of guidelines for treatment, there remains a concern in the medical community about significant overdiagnosis^12^, ^13^, ^14^ and overtreatment.^15^, ^16^ Our exploration of patient attitudes cannot reveal precisely where this bias toward treatment (both biopsy and surgery) originates, but there are clear preferences among some patients for an upfront, surgical approach to remove the risk of cancer growth or metastasis—even when the diagnosis of cancer is uncertain. Interestingly no patient herein explicitly mentioned overtreatment, although several discussed possible misdiagnosis. Previous research indicates that providers are more concerned about overtreatment and overdiagnosis than patients, and that using the word “cancer” prompts patients to want to “get it out.”^16^ This has been demonstrated in large stated preference research in the patients; Nickel et al^17^ noted that respondents who had a lesion described as a “cancer” instead of a “lesion” were significantly more willing to accept lifelong medication and the need to manage hypocalcemia. Recent studies have confirmed similar findings regarding such terminology.^18^ Patient education and the framing of the issue may therefore be determinants of patient choice. Converse to the concept of “getting it out,” several studies indicate that patients who have education about small, low‐risk papillary thyroid carcinoma choose to observe, rather than opt for surgery, in approximately 70% of cases.^19^, ^20^

These seeming contradictions in what patients want, and the tensions between concern for disease progression and concern for treatment‐related side effects, suggest that further large‐scale, quantitative work should be undertaken. Stated preference methods enable both elicitations of underlying patient preferences and the elucidation of the underlying tradeoffs inherent in complex medical decision‐making.^21^, ^22^, ^23^ These techniques have previously been used in otolaryngology and thyroid surgery.^17^, ^24^, ^25^, ^26^, ^27^, ^28^ Studies have demonstrated that antithyroid drugs for Graves disease were generally preferred by patients over surgery or radioactive iodine,^29^ that significant cosmetic considerations regarding thyroid surgery should be factored into decision‐making,^30^ and that risk of needing a subsequent contralateral hemithyroidectomy for those undergoing unilateral procedures is a significant driver of decision‐making.^31^ (Although the topic of hemithyroidectomy was not explicitly addressed in our study, the underlying concerns regarding total vs hemithyroidectomy were explored through discussion of the need for lifelong medication and the risk of potential recurrence.) However, such studies typically describe average preferences across all groups, and there are clear differences within thyroid nodule patients; deeper investigation with latent class analysis into the subgroups and “preference phenotypes” of these patients would more effectively help physicians make personalized decisions regarding care. The relevant attributes identified in our thematic analysis above can assist in this effort.

There are several limitations to this study. While we achieved a purposive sample, we cannot be sure that we have represented all possible themes from the entire population of patients with thyroid disease. Previous studies have shown, however, that there is thematic saturation in this group after approximately 18 patients.^16^ There may be selection bias in those willing to participate in this survey, and it is important to note that 70% of respondents ended up undergoing some sort of surgical treatment (thyroidectomy, lobectomy, completion hemithyroidectomy). Additionally, the setting of research in a surgical clinic may have biased responses; although all responses were confidential and not shared with the treating team, patients may have changed their responses to appeal to their physicians’ opinions. Finally, patients present with varying levels of understanding about their condition and the potential treatment options. While this makes the included population heterogeneous, somewhat limiting generalizability, this heterogeneity is key to capturing a full breadth of opinions, attitudes, and beliefs regarding care, and reflects some of the underlying uncertainty and ambiguity of thyroid nodule management.

## Conclusion

In this thematic analysis of patients with thyroid nodules, patients describe a decision‐making process that incorporates emotional response and rational consideration of risks, contextualized within the personal experience and physician expertise. Patients describe their bias to action and surgical treatment in terms of the unknown risks pertaining to cancer, balanced with metabolic and surgical risks. Ultimately, these findings can help with educational interventions for both physicians and patients and can serve as the backbone for future stated preference research.

## Author Contributions

**Matthew R. Naunheim**, design, conduct, analysis, drafting, final approval; **Manuela von Sneidern**, design, conduct, drafting, approval; **Molly N. Huston**, design, drafting, final approval; **Okenwa C. Okose**, conduct, analysis, drafting, final approval; **Amr H. Abdelhamid** **Ahmed**, design, conduct, final approval; **Gregory W. Randolph**, design, conduct, analysis, drafting, final approval; **Mark G. Shrime**, design, analysis, drafting, final approval.

## Disclosures

### Competing interests

Dr Gregory W. Randolph has received research grants (no personal fees) from Eisai, Medtronic, and Fluoptics, he is the program director of the Massachusetts Eye and Ear Infirmary Endocrine Surgery Clinical Fellowship, which receives partial funding from Medtronic, is the president of the International Thyroid Oncology Group and the World Congress on Thyroid Cancer, is a chair of the Administrative Division of the American Head and Neck Society, and is the American College of Surgeons Otolaryngology Governor. Other authors have no conflicts of interest to report related to the current research.

### Funding source

None.

## Supporting information

Appendix 1. The semi‐structured interview guide.

## References

[1] Dean DS , Gharib H . Epidemiology of thyroid nodules. Best Pract Res Clin Endocrinol Metab. 2008;22(6):901‐911. 10.1016/j.beem.2008.09.019 19041821

[2] Haugen BR , Alexander EK , Bible KC , et al. 2015 American Thyroid Association management guidelines for adult patients with thyroid nodules and differentiated thyroid cancer: the American Thyroid Association guidelines task force on thyroid nodules and differentiated thyroid cancer. Thyroid. 2016;26(1):1‐133. 10.1089/thy.2015.0020 26462967 PMC4739132

[3] Cibas ES , Ali SZ . The Bethesda system for reporting thyroid cytopathology. Am J Clin Path. 2009;132(5):658‐665. 10.1309/AJCPPHLWMI3JV4LA 19846805

[4] Cibas ES , Ali SZ . The 2017 Bethesda system for reporting thyroid cytopathology. Thyroid. 2017;27(11):1341‐1346. 10.1089/thy.2017.0500 29091573

[5] Forner D , Noel CW , Shuman AG , et al. Shared decision‐making in head and neck surgery: a review. JAMA Otolaryngol Head Neck Surg. 2020;146(9):839‐844. 10.1001/jamaoto.2020.1601. 32701131

[6] Naunheim MR , Randolph GW , Shin JJ . Evidence‐based medicine in otolaryngology part XII: assessing patient preferences. Otolaryngol Head Neck Surg. 2020;164:473‐481. 10.1177/0194599820950723 32895002

[7] Preference Collaborative Review Group . Patients' preferences within randomised trials: systematic review and patient level meta‐analysis. BMJ. 2008;337:a1864. 18977792 10.1136/bmj.a1864 PMC2659956

[8] Braun V , Clarke V . Thematic analysis. APA Handbook of Research Methods in Psychology: Research Designs. Vol. 2, ch. 4. American Psychological Association; 2012.

[9] Coast J , Al‐Janabi H , Sutton EJ , et al. Using qualitative methods for attribute development for discrete choice experiments: issues and recommendations. Health Econ. 2012;21(6):730‐741. 10.1002/hec.1739 21557381

[10] Paunonen SV , Jackson DN . The Jackson Personality Inventory and the five‐factor model of personality. J Res Pers. 1996;30(1):42‐59. 10.1006/jrpe.1996.0003

[11] Rampin R , Rampin V . Taguette: open‐source qualitative data analysis. J Open Source Softw. 2021;6(68):3522. 10.21105/joss.03522

[12] Welch HG . Cancer screening, overdiagnosis, and regulatory capture. JAMA Intern Med. 2017;177(7):915‐916. 10.1001/jamainternmed.2017.1198 28492850

[13] Hawkins SP , Jamieson SG , Coomarasamy CN , Low IC . The global epidemic of thyroid cancer overdiagnosis illustrated using 18 months of consecutive nodule biopsy correlating clinical priority, ACR‐TIRADS and Bethesda scoring. J Med Imaging Radiat Oncol. 2021;65(3):309‐316. 10.1111/1754-9485.13161 33665957

[14] Nickel B , Brito JP , Moynihan R , Barratt A , Jordan S , McCaffery K . Patients' experiences of diagnosis and management of papillary thyroid microcarcinoma: a qualitative study. BMC Cancer. 2018;18(1):242. 10.1186/s12885-018-4152-9 29499654 PMC5833084

[15] Higgins S , James BC , Sacks B , Mowschenson P , Nishino M , Hasselgren PO . Can cytologic and sonographic features help prevent overtreatment of Bethesda V thyroid nodules? J Surg Res. 2021;268:112‐118. 10.1016/j.jss.2021.05.050 34298210

[16] Jensen CB , Saucke MC , Francis DO , Voils CI , Pitt SC . From overdiagnosis to overtreatment of low‐risk thyroid cancer: a thematic analysis of attitudes and beliefs of endocrinologists, surgeons, and patients. Thyroid. 2020;30(5):696‐703. 10.1089/thy.2019.0587 31910092 PMC7232663

[17] Nickel B , Howard K , Brito JP , Barratt A , Moynihan R , McCaffery K . Association of preferences for papillary thyroid cancer treatment with disease terminology: a discrete choice experiment. JAMA Otolaryngol Head Neck Surg. 2018;144(10):887‐896. 10.1001/jamaoto.2018.1694 30140909 PMC6233835

[18] Dixon PR , Tomlinson G , Pasternak JD , et al. The role of disease label in patient perceptions and treatment decisions in the setting of low‐risk malignant neoplasms. JAMA Oncol. 2019;5(6):817‐823. 10.1001/jamaoncol.2019.0054 30896738 PMC6567830

[19] Sawka AM , Ghai S , Yoannidis T , et al. A prospective mixed‐methods study of decision‐making on surgery or active surveillance for low‐risk papillary thyroid cancer. Thyroid. 2020;30(7):999‐1007. 10.1089/thy.2019.0592 32126932 PMC7374636

[20] Sawka AM , Ghai S , Rotstein L , et al. A quantitative analysis examining patients' choice of active surveillance or surgery for managing low‐risk papillary thyroid cancer. Thyroid. 2022;32(3):255‐262. 10.1089/thy.2021.0485 35019770

[21] Bridges JF . Stated preference methods in health care evaluation: an emerging methodological paradigm in health economics. Appl Health Econ Health Policy. 2003;2(4):213‐224. 15119540

[22] Naunheim MR , Wittenberg E , Shrime MG . Patient preference research in otolaryngology: what do patients want? JAMA Otolaryngol Head Neck Surg. 2017;143:971. 10.1001/jamaoto.2017.1086 28727861

[23] O'Hara NN . Eliciting health care preferences with discrete choice experiments. JAMA Netw Open. 2022;5(4):e228794. 10.1001/jamanetworkopen.2022.8794 35467737

[24] Grutters JPC , Joore MA , Kessels AGH , Davis AC , Anteunis LJC . Patient preferences for direct hearing aid provision by a private dispenser. A discrete choice experiment. Ear Hear. 2008;29(4):557‐564. 10.1097/AUD.0b013e3181734a19 18469716

[25] Tankersley M , Winders T , Aagren M , et al. Preference for immunotherapy with tablets by people with allergic rhinitis. Patient Prefer Adherence. 2021;15:2539‐2549. 10.2147/PPA.S338337 34819723 PMC8608245

[26] Naunheim MR , Naunheim ML , Rathi VK , Franco RA , Shrime MG , Song PC . Patient preferences in subglottic stenosis treatment: a discrete choice experiment. Otolaryngol Head Neck Surg. 2018;158(3):520‐526. 10.1177/0194599817742851 29161192

[27] Fischman V , Wittenberg E , Song SA , et al. How patients choose a laryngologist: a pilot stated preference study. OTO Open. 2021;5(1):2473974X21999601. 10.1177/2473974X21999601 PMC7968048 33796810

[28] Naunheim MR , Rathi VK , Naunheim ML , et al. What do patients want from otolaryngologists? A discrete choice experiment. Otolaryngol Head Neck Surg. 2017;157(4):618‐624. 10.1177/0194599817717662 28675119

[29] van Kinschot CMJ , Soekhai VR , de Bekker‐Grob EW , et al. Preferences of patients and clinicians for treatment of Graves’ disease: a discrete choice experiment. Eur J Endocrinol. 2021;184(6):803‐812. 10.1530/EJE-20-1490 33780350

[30] Sukpanich R , Sanglestsawai S , Seib CD , et al. The influence of cosmetic concerns on patient preferences for approaches to thyroid lobectomy: a discrete choice experiment. Thyroid. 2020;30(9):1306‐1313. 10.1089/thy.2019.0821 32204688

[31] Ahmadi S , Gonzalez JM , Talbott M , et al. Patient preferences around extent of surgery in low‐risk thyroid cancer: a discrete choice experiment. Thyroid. 2020;30(7):1044‐1052. 10.1089/thy.2019.0590 32143553

