# Supplementary material for: Patient Experiences With Thyroid Nodules: A Qualitative Interview Survey
Source: OTO Open. 2023 Mar 8;7(1):e39. doi: 10.1002/oto2.39 (PMC10046705; doi:10.1002/oto2.39)
Supplement: Supplementary file 1 — Appendix 1. The semi‐structured interview guide. [file OTO2-7-e39-s001.docx]

**Appendix 1. Semi-structured interview guide for thyroid nodule patients.**

1. What were your initial thoughts when you were told you may have a thyroid nodule? What comes to mind?
   1. [If the patient does not have any responses, please try these prompts]
      1. Did you have any emotional feelings?
      2. Did it have you thinking about the future?
      3. Have any family members had this problem or a similar one?
2. How important do you think it is to have your thyroid nodule treated?
   1. [If the patient does not have any responses, please try these prompts]
      1. More important than yearly checkup with primary doctor?
      2. More important than screenings, like those for prostate or breast cancer? (by gender)
3. Was your treatment decision a hard decision for you to make? If so, why? If not, why not?
   1. [If the patient does not have any responses, please try these prompts]
      1. How did you make it?
4. Tell me your concerns about getting NO treatment. In other words, what would you worry about if you were observed periodically, but no treatment were done?
   1. [If the patient does not have any responses, please try these prompts]
      1. Are you a worrier in general? What does worry you?
5. Tell me your concerns about getting SURGICAL treatment. In other words, what would you worry about if you decided you wanted surgery for this?
   1. [If the patient does not have any responses, please try these prompts]
      1. Think about past surgeries. What were your worries then?
6. How concerned are you about side effects of treatment? Which ones?
7. How concerned are you about the risk of cancer?
8. Have you ever talked about your thyroid problems with any friends or family? Did this help? How? Do you think this helped you?
   1. [If the patient does not have any responses, please try these prompts]
      1. Are you a private person in general?
      2. What would you tell your family/friend if they were here right now?
9. Please tell me which of the following things affected your decision. Please answer yes or no to the following questions. Did you think about:
   1. Risk of developing cancer?
   2. Need for many follow up visits for surveillance
   3. Fear of not knowing what the nodule is
   4. Fear of having a needle biopsy
   5. Risk of undergoing surgery
   6. Concern about surgical complications
   7. Concern for treatment side effects
   8. Concern about pain
   9. Effectiveness of treatment
   10. Need for multiple treatments in the future
   11. Need for the most definitive treatment
   12. Not wanting to stay overnight in the hospital
   13. Cost of the treatment / coverage by insurance
   14. Your doctor’s opinion
   15. Your friend and family’s opinions
   16. Which of these things was MOST important to you?
10. How comfortable do you feel making a decision on your own about what to do for your thyroid problem? Do you have strong opinions, or would you rather listen to the doctor’s advice?
11. Suppose you were trying to help a friend to decide what to do about a thyroid nodule… What would you say?
12. What did you decide to do with your thyroid nodule?
13. Finally, I will ask you to respond to three general statements.
    1. “Taking risks does not bother me if the gains involved are high”
       1. Do you disagree strongly, disagree somewhat, feel neutral, agree somewhat, agree strongly, or are you not sure?
    2. “I enjoy taking risks”
       1. Do you disagree strongly, disagree somewhat, feel neutral, agree somewhat, agree strongly, or are you not sure?
    3. “People have told me that I seem to enjoy taking chances”
       1. Do you disagree strongly, disagree somewhat, feel neutral, agree somewhat, agree strongly, or are you not sure?
